# Electroacupuncture activates the peroxisome proliferators‐activated receptor pathway to improve the phenotype of cerebral palsy

**DOI:** 10.1111/cns.14876

**Published:** 2024-07-25

**Authors:** Zhi‐Feng Wu, Hong‐Hao Peng, Yun Shu, Li Zhang, Si Zhang, Jing‐Yang Zhang, Si‐jie Li, Qiong‐Li Fan, Yun Wei, Li Ming, Jing‐Jing Tong, Yu‐Ping Zhang

**Affiliations:** ^1^ Department of Pediatrics Army Medical University Xinqiao Hospital Chongqing China; ^2^ Medical College of Acu‐Moxi and Rehabilitation Guangzhou University of Chinese Medicine Guangzhou China; ^3^ Department of Neurosurgery Children's Hospital of Chongqing Medical University, National Clinical Research Center for Child Health and Disorders, Ministry of Education Key Laboratory of Child Development and Disorders, Chongqing Key Laboratory of Pediatrics Chongqing China; ^4^ Department of Radiology Army Medical University Xinqiao Hospital Chongqing China; ^5^ Department of Traditional Chinese Medicine Army Medical University Xinqiao Hospital Chongqing China

**Keywords:** cerebral palsy, electroacupuncture, PPAR pathway, rosiglitazone

## Abstract

**Aim:**

This study explores the efficacy of electroacupuncture (EA) in treating cerebral palsy (CP) in Sprague–Dawley (SD) pups, specifically CP animal models, and its molecular mechanisms.

**Methods:**

Gait analysis and Y‐maze were used to detect the improvement of motor ability and cognitive function of CP rats after EA treatment. Transcription sequencing was used to determine the key pathway for EA to improve the symptoms of CP. PPAR agonists were used to verify the causal relationship between the pathway and the improvement of CP phenotype.

**Results:**

The motor ability and cognitive function of CP pups were improved after EA treatment. The results of transcriptome sequencing suggest that the improvement of CP phenotype may be caused by the activation of PPAR pathway. PPAR pathway is widely activated in the epithelium of CP pups treated with EA, which is verified by qPCR. Rosiglitazone (Ros), a PPAR agonist, can improve CP phenotype while activating PPAR pathway, which proves the causal relationship between PPAR pathway activation and CP phenotype improvement.

**Conclusion:**

Our study demonstrated behavioral improvements and enhanced cognitive functions in CP models after EA treatment by activating PPAR pathway, suggesting new perspectives for CP rehabilitation, and providing theoretical support for acupuncture treatment of CP.

## Graphical abstract

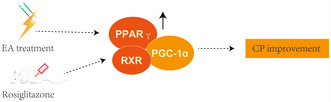

## 1 INTRODUCTION

Cerebral Palsy (CP), a syndrome characterized by ongoing central motor and postural developmental impairments leading to restricted activities, is caused by non‐progressive brain damage in fetuses or infants during development.^1^ This condition is a major cause of disability in children, severely affecting their physical and mental health.^2^, ^3^ With the improvement of obstetric techniques and the advancement of perinatal and neonatal medicine, survival rates for high‐risk and very low‐birth‐weight infants have significantly increased. However, the incidence of CP is showing a steady and concerning rise.^4^ Around 2‰ of live births worldwide are diagnosed with CP, with a prevalence of 2.3‰ among children aged 0–6 in China,^5^ affecting over 5 million individuals. The causes of CP are varied and its mechanisms extremely intricate.^6^ Clinical treatment often involves a combination of drugs, surgery, exercise, and physical therapy.^7^ However, these treatments are characterized by long duration, suboptimal outcomes, high costs, and significant time and effort investment, making the treatment of CP in children a focal point and challenge in clinical practice.^8^ This highlights the pressing need for the discovery of new treatment methods and the development of more efficient drugs.

In the traditional medical treatment of CP, neurostimulation via acupuncture is preferred.^9^, ^10^ Electroacupuncture (EA), an advancement on traditional acupuncture, offers precise control of the stimulation intensity, facilitating standardized, and regulated procedures.^11^ This study utilized the “International Standard of Scalp Acupuncture Points,” targeting the “Anterior Oblique Line of Vertex‐Temporal (DǐngnièQiánxiéxiàn, MS6)” for children with CP. According to anatomical structure, MS6 corresponds to the motor cortex under the skull, traditionally treating motor disorders in stroke and encephalopathy.^12^, ^13^, ^14^ However, current understanding lacks mechanistic studies on physiological or pathological effects post‐MS6 stimulation.

Despite extensive practice in China, the unclear mechanism of acupuncture for CP impedes global adoption.^15^ Therefore, enhancing the research into the mechanisms of acupuncture in treating CP is crucial for elevating the scientific standards of its application in CP and to help develop potential ways to improve chronic neurological conditions and help children with cerebral palsy achieve better development. This study aims to employ transcriptomics technology to uncover the molecular mechanisms of EA in treating pups with CP and to verify the causal relationship between these molecular mechanisms and behavioral improvements in CP pups. Such research will clarify the molecular mechanisms underlying EA treatment for CP, offering new perspectives for the rehabilitation of children with CP, and providing theoretical support for clinical treatment of CP with EA.

## 2 METHODS

### 2.1 Animals experimental design and implementation plan

All experimental protocols were approved by the institutional animal care and use committee (IACUC) at Army Medical University (AMUWEC20234543) and were in accordance with the guidelines published in the National Institutes of Health Guide for the Care and Use of Laboratory Animals. All manipulations during the experiment met the requirements for animal welfare. In adherence to the 3R principles (Replacement, Reduction, and Refinement) for animal experimentation and statistical requirements, our study designated a group size of six animals. Accounting for an anticipated 20% attrition rate due to factors such as mortality, a total of 94 pups were utilized in this research.

Ninety‐four healthy Sprague–Dawley (SD) pups at postnatal day 10, obtained from the Animal Experiment Center of Army Military Medical University, irrespective of gender, were selected and divided into two batches for purchasing and experimentation. The standard = RAND() function in Microsoft Excel was utilized for random selection and grouping in this study.

**FIGURE 1:**
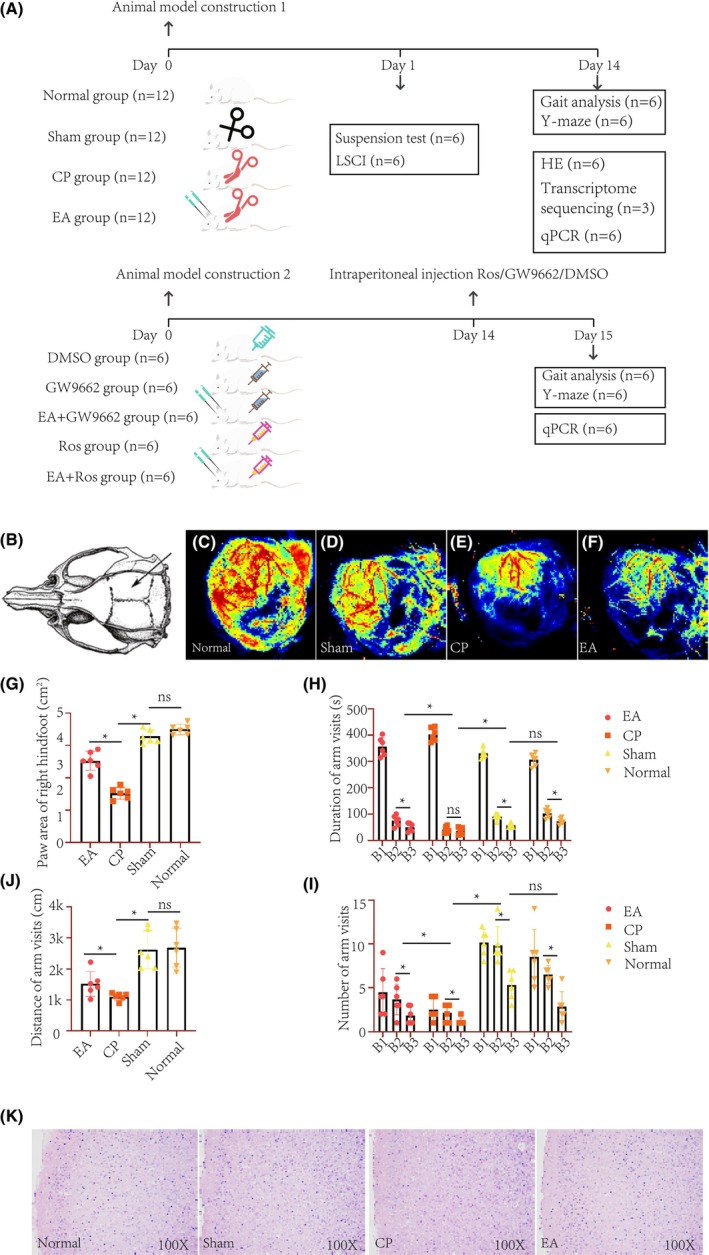
Immediate electroacupuncture treatment enhances motor and cognitive functions in cerebral palsy rats. (A): Experimental design of the study. The study was divided into two parts. In the first part, 48 rats were randomly categorized into four groups: Normal, sham surgery, cerebral palsy, and electroacupuncture treatment, with 12 rats in each group. One day after modeling, half of the pups in each group underwent suspension experiments and laser speckle contrast imaging (LSCI), and in order to rule out the impact of LSCI surgery on subsequent experiments, these pups did not participate in the follow‐up experiments. The remaining rats underwent gait analysis, Y‐maze tests, Hematoxylin and Eosin (HE) staining, and qPCR validation after 2 weeks of electroacupuncture treatment. Motor cortex from three rats each in the electroacupuncture and cerebral palsy groups were selected for transcriptomic sequencing. In the second part, 30 CP rats were randomly divided into five groups with six in each group: DMSO, GW9662, EA + GW9662, ROS, and EA + ROS. Following 2 weeks of electroacupuncture treatment, rats received intraperitoneal injections of different drugs according to their group, followed by gait analysis, Y‐maze tests, and qPCR validation 24 h later. (B–F): Cerebral blood flow detection regions and laser imaging maps in rats. (G): Gait analysis; (H–I): Y‐maze tests, B1: Start arm; B2: Novel arm; B3: Other arm (*p < 0.05; **p < 0.01). (K): Hematoxylin and Eosin (HE) staining.

For the first batch, 56 postnatal day 10 SD pups were purchased. Among these, 12 pups were allocated to the normal group, while 13 pups were assigned to the sham group, where pups underwent a neck incision without common carotid artery ligation. After anesthesia recovery, one pup was randomly excluded, leaving 12 pups in the sham group. Thirty‐one pups underwent Rice‐Vanucci modeling, with 26 pups surviving after anesthesia recovery and reflex confirmation, yielding an 84% modeling success rate. Two pups were randomly excluded, leaving 24 CP pups, randomly divided into CP control (*n* = 12) and EA (electroacupuncture) groups (*n* = 12), Figure 1A. In the first batch of experiments, there were four groups with 12 rat pups each. After recovery from day 1, six rat pups from each group were randomly selected using the RAND() function in Excel for suspension test and laser speckle contrast imaging (LSCI). Due to the need for anesthesia and scalp incision for LSCI (refer to the LSCI method section), these six rat pups from each group were excluded from subsequent experiments to avoid potential interference with further testing. The remaining six rat pups in each of the four groups continued with subsequent experiments.

For the second batch, 38 postnatal day 10 SD rat pups were purchased. After Rice‐Vanucci modeling and recovery, 33 pups survived and confirmed successful modeling, resulting in an 86.8% success rate. Three pups were randomly excluded, leaving 30 pups, randomly distributed into DMSO (*n* = 6), GW9662 (*n* = 6), EA + GW9662 (*n* = 6), Ros (*n* = 6), and EA + Ros (*n* = 6) groups, Figure 1A. Prior to group allocation and treatment, it's important to note that Rosiglitazone (Ros) is a peroxisome proliferator‐activated receptor (PPAR) agonist activator, while GW9662 is a PPARγ‐specific antagonist. The DMSO group was used as a control group with the same DMSO concentration as the other experimental groups.

### 2.2 CP molding surgery

The CP molding surgery followed the classic Rice‐Vanucci model,^16^ the pups are anesthetized by ether inhalation, ensuring spontaneous breathing during surgery. After anterolateral neck incision and disinfection, the left common carotid artery was isolated and ligated with a double silk thread. The rectal temperature of the pups was maintained between 36.5°C and 37.5°C during surgery. Postoperatively, animals were housed in an environment with 8% oxygen and 92% nitrogen (temperature controlled at 34°C, humidity at 85 ± 5%) for 1 h. After hypoxic housing, the animals were transferred to a ventilated and air‐conditioned animal room. The righting reflex disappeared during waking after surgery, and spontaneous right‐handed or clipped right‐handed was used in follow‐up experiments in pups. The sham operation group underwent only a neck incision without ligation of the common carotid artery, causing no animal damage. The normal group received neither surgery nor hypoxia exposure.

### 2.3 Electroacupuncture treatment

The CP pups underwent EA therapy targeting the head motor area known as the “Anterolateral Oblique Line of the Vertex‐Temporal (MS6).”^17^ The treatment procedure involved the careful subcutaneous insertion of sterile disposable acupuncture needles (0.25 × 13 mm) into the bilateral acupoints MS6. The depth of insertion was visually confirmed and set at 3/5 of the distance from Shenchong (Exhn1) to Xuanli (GB06).

The needles were then connected to an EA instrument (805‐A, Dajia, China) set at a continuous wave frequency of 10 Hz. The current intensity was meticulously adjusted at 1 mA to ensure appropriate level for juvenile pups. Each treatment session lasted for 5 min, and the pups were closely monitored for any signs of discomfort or distress. In the event of such indications, the procedure was promptly discontinued. The EA therapy was administered once daily for a total of 14 days.

To maintain uniformity across the groups, all groups were subjected to an identical fixation method and duration (EA group received 5 min of EA treatment, while the remaining groups were also secured for a comparable 5 min period) (refer to Figure S1). All treatments were administered by the same operator, following strict protocols for randomization using a RAND function in Microsoft Excel (Microsoft Excel 2023), and ensuring blinding through double‐blind trials. EA treatment commenced on the second day post‐modeling and continued for a duration of two weeks (Figure 1A).

### 2.4 Suspension test

The forelimbs of the pups were allowed to grasp a horizontal plastic rod, 0.3 cm in diameter and 45 cm above the tabletop. The time until the pups fell was recorded. The scoring criteria for the suspension test were as follows: 0 points for hanging 0–5 s; 1 point for 6–10 s; 2 points for 11–15 s; 3 points for 16–20 s; 4 points for 21–25 s; 5 points for 26–30 s; 6 points for more than 30 s.

### 2.5 Laser speckle contrast imaging (LSCI)

In the laboratory, the Laser Speckle Imaging System (RFLSI ZW, Reward) was used. Rats were anesthetized with pentobarbital (50 mg/kg) via intraperitoneal injection, with additional anesthesia boosters (1/3 of the initial dose) administered as needed. Following anesthesia, the rat pups' scalps were mid‐sagittally incised, and the skull membranes were gently peeled off using a cotton swab, while ensuring minimal disruption to the surface vasculature. The laser was evenly projected vertically onto the pup's skull, illuminating an approximately 5 cm spot to cover the entire cranial area. Backscattered light signals from the skull were captured by a CMOS Global Shutter Camera with a resolution of 2064*1544 pixels. Raw speckle images were processed in real‐time online with a laser speckle blood flow algorithm, converting them directly into blood perfusion information. The imaging software used was the online laser blood flow imaging system.

### 2.6 Gait analysis

Before gait analysis, pups were adaptively trained in the animal gait analyzer (DigiGait, Reward) for 3 days. Gait analysis was conducted for each group of pups before tissue separation. The analyzer automatically generated the maximum contact area of the right hind paw for analysis.

### 2.7 Y‐maze

The spatial exploration, memory, and motor abilities of juvenile pups were evaluated using a Y‐shaped maze apparatus (Reward). The Y‐maze consisted of three arms set at 120‐degree angles to each other, with each arm measuring 30 cm in length, 8 cm in width, and 15 cm in height. A camera was mounted at the top center to record the exploratory behavior of the pups. The three arms were designated as the start arm, novel arm, and other arm. During the training phase, the novel arm was blocked, and pups were placed in the start arm, allowing them to freely explore between the start and other arms for 10 min. Afterwards, 10% alcohol was used to wipe and dry the start and other arms to eliminate olfactory cues from affecting subsequent experiments. After a 5 min interval, the testing phase commenced with the blockade of the novel arm removed. Pups were then placed in the start arm of the maze, permitting free exploration across all three arms for 8 min, with their activity being videotaped. The results of the Y‐maze experiment were analyzed using EthoVision XT 15 software (Noldus, Netherland). The primary analytical metrics included calculating the cumulative time spent by the pups in the novel arm, the frequency of entries into the novel arm, and the total path length covered.

### 2.8 Hematoxylin and eosin staining

After rapid decapitation following intraperitoneal anesthesia of the animals, the brain tissue was extracted on an ice‐cold plate. The bilateral cortical tissues were then fixed, dehydrated, cleared, and embedded in paraffin for paraffin sectioning. Hematoxylin and eosin (HE) staining was performed to assess the pathological condition of the lesioned left cerebral cortex.

### 2.9 Real‐time quantitative PCR

Total RNA from each group of pups was extracted using the UNlQ‐10 Column Trizol Total RNA Isolation Kit (#511321–0100, Sangon Biotech, China) and converted to cDNA using the M‐MuLV First Strand cDNA Synthesis Kit (#532435–0100, Sangon Biotech). Excess RNA was stored in a − 80°C ultra‐low‐temperature freezer (Haier Biomedical), while surplus cDNA was kept at −20°C. For the qPCR reaction, 2X SG Fast qPCR Master Mix (#B639271‐0005, Sangon Biotech) and cDNA samples was prepared on a qPCR plate (#GV‐PCR‐0108‐C, GENVIEW), with appropriate denaturation, annealing, and extension times and temperatures set on the qPCR instrument CFX Connect (Bio‐Rad). Samples were sourced from the left cerebral cortex of rat pups.

### 2.10 Transcriptome sequencing

In order to investigate the molecular mechanisms underlying the therapeutic effects of EA on motor impairment resulting from left common carotid artery ligation, three rat pups each from the CP group and EA group were randomly selected for transcriptome sequencing of samples obtained from the left cerebral cortex. The sequencing data were processed and analyzed using R Studio (version 4.3.0), with the criteria for differential expression analysis set as log fold change (logFC) >2 and adjusted *p*‐value <0.05. KEGG pathway enrichment analysis was conducted using the web‐based tool David (david.ncifcrf.gov).

### 2.11 Peroxisome proliferators‐activated receptor agonists/inhibitors treatment

In the second part of our experiment, we investigated the effects of PPAR agonists/inhibitors in a CP model involving 30 pups (Figure 1A animal model construction 2). These pups were randomly divided into five equal groups. The treatment regimens were as follows: ①DMSO (#A100231‐0500, Sangon Biotech) Group: CP model SD pups received an intraperitoneal injection of 20% DMSO as a pretreatment. ②GW9662 (#HY‐16578, MCE) Group: CP model SD pups received an intraperitoneal injection of the PPAR pathway inhibitor GW9662 at a dose of 0.2 mg/kg. ③EA + GW9662 (#HY‐16578, MCE) Group: Post‐EA treatment, CP model SD pups received an intraperitoneal injection of the PPAR pathway inhibitor GW9662 at a dose of 0.2 mg/kg. ④Rosiglitazone (Ros) (#HY‐17386, MCE) Treatment Group: CP model SD pups were treated with an intraperitoneal injection of the PPAR pathway agonist Ros at a dose of 0.5 mg/kg. ⑤EA + Ros Group: Following EA treatment, CP model SD pups were administered an intraperitoneal injection of the PPAR pathway agonist Ros at a dose of 0.5 mg/kg.

### 2.12 Statistical analysis

The Kolmogorov–Smirnov test was utilized to assess the distributional properties of the datasets. Specifically, the Suspension test, blood perfusion from Laser speckle imaging, Gait analysis, and Y‐maze data underwent Kolmogorov–Smirnov tests to assess normality. All datasets exhibited a normal distribution, confirming the suitability for parametric analyses. Subsequently, group comparisons were conducted using one‐way analysis of variance (ANOVA) with the Bonferroni or Games‐Howell method, while paired‐samples *t*‐tests were employed for within‐group comparisons. Results were reported as mean ± standard deviation (SD) derived from a minimum of three independent experiments. A significance threshold of *p* < 0.05 was consistently maintained across all statistical analyses.

## 3 RESULTS

### 3.1 Electroacupuncture improved the symptoms of the cerebral palsy pups model

The test protocol was shown in Figure 1A (Figure 1A). Twenty‐four hours post CP modeling, a significant reduction in suspension time was observed in CP model pups compared to the sham‐operated group (*p* < 0.05). However, the suspension time in the sham‐operated group showed no significant difference compared to normal SD pups (*p* > 0.05). An immediate effect of a single session of EA treatment was noted, partially restoring and prolonging the suspension time in CP pups (*p* < 0.05), indicating successful CP modeling (Table 1). Laser speckle imaging experiments revealed that 24 h after ligation of the left common carotid artery, the cortical blood flow in the left cerebral hemisphere decreased to approximately 51.23% of pre‐ligation levels (*p* < 0.01). Post a single EA treatment session, an increase in 20.48% in left cerebral cortical blood flow was observed compared to post‐ischemic levels (*p* < 0.01). No significant change in cortical blood flow was noted in the sham‐operated group compared to the normal group (*p* > 0.05) (Figure 1B–F and Table 2). These experiments not only confirmed the successful construction of the CP pup model but also demonstrated the immediate effect of a single EA treatment in promoting blood flow reperfusion and extending suspension time in CP pups.

**TABLE 1:** Comparison of the results of rats suspension test ($\bar{x}\pm s$).

Group	Number	Suspension test score	*p* value
CP	6	0.98 ± 0.17^a^	<0.01
EA	6	3.71 ± 0.07^b^	<0.01
Sham	6	5.17 ± 0.41^c^	0.50
Normal	6	5.31 ± 0.35	/

*Note*: Data collected for the number of rats in each group was obtained after one EA intervention or an equivalent fixation method, and analyzed using ANOVA.

^a^ The suspension score of CP group was significantly lower than that of sham operation group.

^b^ The suspension score of EA group was significantly higher than that of CP group.

^c^ No differences detected between the Sham and Normal groups.

**TABLE 2:** Comparison of blood perfusion between left and right hemisphere ($\bar{x}\pm s$).

Group	Number	Blood perfusion(L)	Blood perfusion(R)	*p* value
CP	6	407.09 ± 1.18	794.7 ± 4.22	<0.01
EA	6	442.66 ± 38.4	617.31 ± 33.98	<0.01
Sham	6	812.59 ± 49.58	787.93 ± 69.37	0.07
Normal	6	796.36 ± 8.74	795.97 ± 6.54	0.10

*Note*: Data collected for the number of rats in each group was obtained after one EA intervention or an equivalent fixation method, and analyzed using paired‐samples *t*‐test. The unit of blood perfusion is PU.

At the 2‐week post‐treatment mark, a comparison with the sham‐operated group revealed a decrease in the maximum contact area of the right hind paw in the CP group (*p* < 0.05). No significant difference was observed between the sham‐operated group and normal pups (*p* > 0.05). In contrast, the EA treatment group exhibited an increase in the maximum contact area of the right hind paw compared to the CP group (*p* < 0.05) (Figure 1G).Compared to the sham‐operated group, the CP group pups spent significantly less time in the novel arm of the Y‐maze (*p* < 0.05); post EA treatment, the duration increased significantly (*p* < 0.05, Figure 1H). Furthermore, the number of entries into the novel arm was significantly fewer in the CP group compared to other arms (*p* < 0.05); this number significantly increased following EA treatment (*p* < 0.05, Figure 1I). The total distance traveled in the Y‐maze within 8 min was significantly lower in the CP group compared to the sham‐operated group (*p* < 0.05); however, it was significantly higher in the EA treatment group compared to the CP group (*p* < 0.05, Figure 1J). In the control and sham‐operated groups, cortical cells appeared full, tightly arranged, and structurally clear. In contrast, CP group exhibited sparse and disordered cortical cell arrangement, reduced cell bodies, and evidence of nuclear fragmentation; these pathological symptoms were significantly alleviated in the EA treatment group (Figure 1K).

### 3.2 Electroacupuncture activated the peroxisome proliferators‐activated receptor pathway to improve the symptoms of pup model with cerebral palsy

**FIGURE 2:**
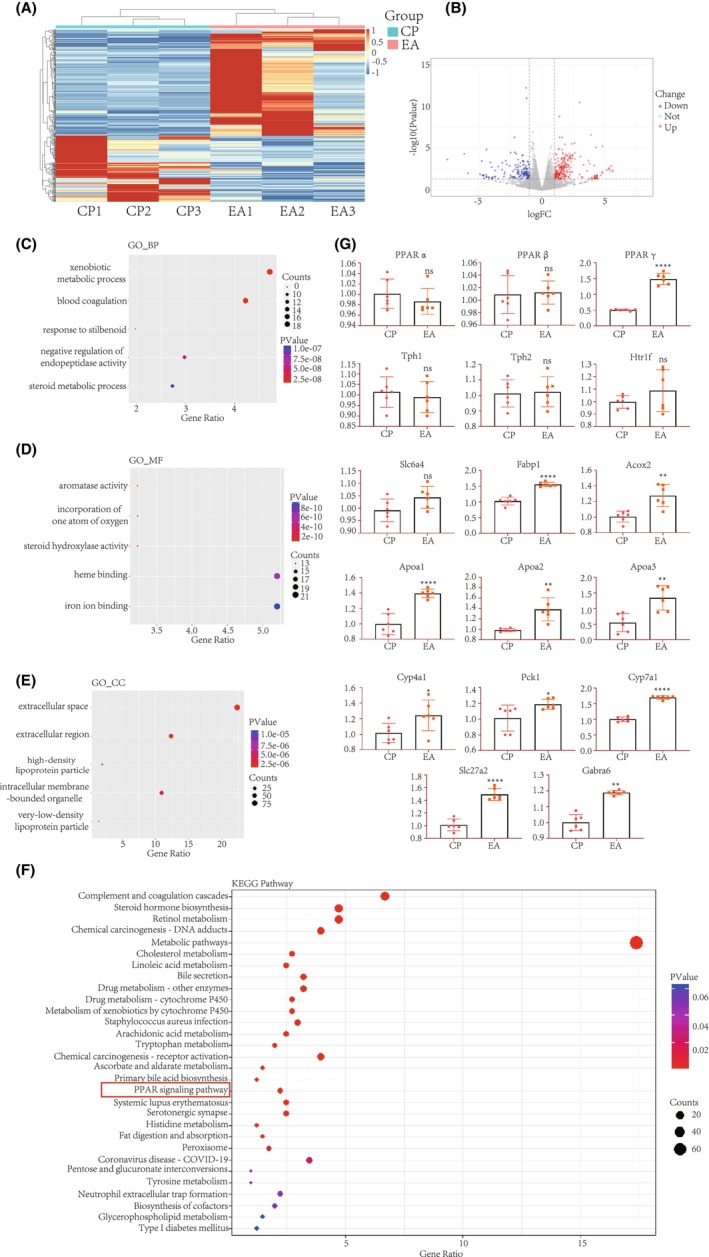
Potential pathways of electroacupuncture‐induced improvement in cerebral palsy phenotypes in rats via PPAR pathway activation. (A,B): Cluster analysis and volcano plots of differentially expressed genes from transcriptome sequencing. (C–F): Results of Gene Ontology (GO) and Kyoto Encyclopedia of Genes and Genomes (KEGG) pathway enrichment analyses from transcriptome sequencing. (G): QPCR validation of the serotonergic synaptic pathway, PPAR pathway, and Gabra6 gene. *Tph1*, *Tph2*, *Htr1f*, and *Slc6a4* are part of the serotonergic synaptic pathway, while the remaining genes belong to the PPAR pathway (*p < 0.05; **p < 0.01; ****p < 0.0001).

Differential expression analysis identified 407 genes with *p* < 0.05 and log2 Fold Change Index (FCI) >1 as differentially expressed genes (DEGs) between the EA and CP groups. Among these, 156 genes were downregulated, and 251 were upregulated (Figure 2A,B). Gene Ontology (GO) enrichment analysis yielded a total of 278 terms, comprising 190 Biological Process (BP) terms, 64 Molecular Function (MF) terms, and 24 Cellular Component (CC) terms. Results for each term were ranked by significance, with the top five results from each category displayed.

Analysis using the GO database revealed major enriched results in the BP category, including “xenobiotic metabolic process,” “blood coagulation,” “response to stilbenoid,” “negative regulation of endopeptidase activity,” and “steroid metabolic process” (Figure 2C). In the MF category, significant enrichments included “aromatase activity,” “oxidoreductase activity,” “steroid hydroxylase activity,” “heme binding,” and “iron ion binding” (Figure 2D). In the CC category, the main enrichments were “extracellular space,” “extracellular region,” “high‐density lipoprotein particle,” “intracellular membrane‐bounded organelle,” and “very‐low‐density lipoprotein particle” (Figure 2E). KEGG pathway enrichment analysis identified 35 terms, with brain‐related pathways including “Serotonergic synapse” and “PPAR signaling pathway” (Figure 2F). Quantitative PCR (qPCR) validation was performed for genes related to the PPAR pathway and serotonergic synapse (Figure 2G). The results indicated widespread activation of genes associated with the peroxisome proliferator‐activated receptor (PPAR) pathway, suggesting its potential significant role in the EA treatment of CP.

### 3.3 Treated with the peroxisome proliferators‐activated receptor agonist Ros demonstrated a causal relationship between peroxisome proliferators‐activated receptor pathway activation and improvement in cerebral palsy symptoms

**FIGURE 3:**
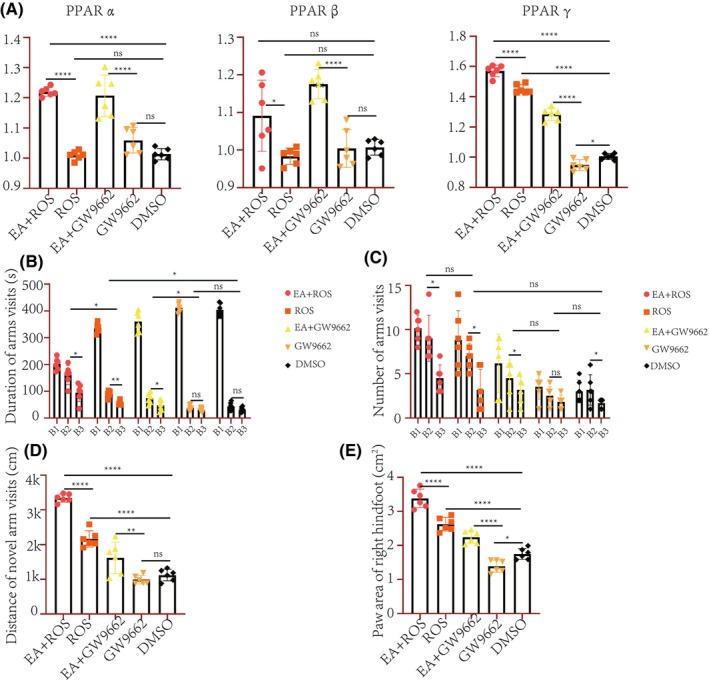
Intraperitoneal injection of rosiglitazone and GW9662 confirms the causal relationship between PPAR pathway activation and improvement in cerebral palsy phenotypes in rats. (A): RNA expression levels of *PPARα*, *PPARβ*, and *PPARγ* in rats from the DMSO, GW9662, EA + GW9662, ROS, and EA + ROS groups. (B–D) Y‐maze tests, B1: Start arm; B2: Novel arm; B3: Other arm. (E): Gait analysis (*p < 0.05; **p < 0.01; ***p < 0.001; ****p < 0.0001).

To further investigate whether EA ameliorates CP symptoms via the PPAR pathway, we employed Rosiglitazone (Ros) as a PPAR agonist and GW9662 as a PPARγ inhibitor. Figure 3A illustrates that Ros significantly upregulated genes associated with the PPAR pathway both inter‐ and intra‐group, whereas the PPAR pathway inhibitor GW9662 downregulated these genes' expression. Notably, EA supplementation under identical treatment conditions resulted in statistically significant differences compared to their respective non‐EA counterparts (EA + Ros vs. Ros, EA + GW9662 vs. GW9662), detailed in Figure 3A.

Intra‐group analysis revealed that both the Ros group (PPAR agonist) and the combined EA and Ros treatment group spent a markedly longer duration in the novel arm relative to other arms (*p* < 0.05). Conversely, no significant difference was observed in the duration spent in the novel arm among the GW9662 or DMSO groups (negative control, Figure 3B). Upon inter‐group comparison, the combined EA and Ros treatment group displayed a prolonged duration in the novel arm compared to the Ros groups. The Ros group outperformed the DMSO group. It is noteworthy that all groups incorporating EA outperformed their non‐EA counterparts under equivalent conditions (*p* < 0.05), while the GW9662 group underperformed relative to the DMSO group. These observations imply that both EA treatment and Ros administration may enhance spatial memory capabilities in hypoxic ischemic pups (see Figure 3B). Additionally, upon examining the number of entries into each arm by the pups within each group, the EA + GW9662 group, Ros group, and the combined EA and Ros treatment group made significantly more entries into the novel arm compared to other arms (Figure 3C). Investigation into the total distance covered within 480 seconds revealed that the combined EA and Ros treatment group exhibited superior exploration of the novel arm compared to both the Ros and DMSO groups. While EA + GW9662 showed better performance than the GW9662 group, the combined EA and Ros treatment cohort exhibited the most favorable performance (*p* < 0.01, as depicted in Figure 3D).

Gait analysis revealed that the combined EA and Ros treatment group outperformed the Ros group, which in turn showed enhanced outcomes relative to the DMSO group with respect to the right hind paw print area (*p* < 0.05). Furthermore, the EA + GW9662 group exhibited a larger paw area compared to the GW9662 group, collectively endorsing the superior efficacy of the EA + Ros regimen (Figure 3E). These results imply that EA treatment, PPAR pathway activation, or their combined application may enhance cognitive function and mobility in a hypoxic–ischemic model.

## 4 DISCUSSION

Cerebral palsy (CP) primarily manifests as motor impairments, often accompanied by sensory, perceptual, and cognitive deficits. Currently, the management of CP in the clinic remains a significant medical challenge. Clinical management typically integrates various interventions such as activities of daily living training, goal‐directed training (GDT), multisensory stimulation, games, physical therapy, occupational therapy, and family interventions. In China, alongside these clinical interventions, traditional acupuncture is favored, with recent advancements like painless acupuncture gaining popularity among pediatric patients.^18^ Globally, pediatric acupuncture has shown significant growth. The prevalence of pediatric acupuncture users rose from 1.78% in 2002 to 5.34% in 2011 (*p* < 0.0001). Factors such as older age, urban residency, and musculoskeletal disorders correlate with higher acceptance of acupuncture. Notably, cerebral palsy and psychoses were predominant among those receiving complex acupuncture treatments.^15^ Based on anatomical structure and principles from traditional Chinese medicine, MS6 located on the scalp emerges as the predominant acupuncture point in clinical practice. Therefore, our study has specifically chosen MS6, the clinically favored point, for treating the rat model of CP.^12^, ^13^, ^14^ A meta‐analysis,^19^ encompassing 11 studies with 731 children (369 in the experimental group and 362 controls), indicated that scalp acupuncture markedly ameliorated symptoms in CP patients, enhanced psychological development, and improved motor abilities. Further randomized controlled trials,^20^ demonstrated that scalp acupuncture effectively treats spastic CP by enhancing cerebral hemodynamics, gross motor functions, and daily living activities, while reducing muscle tension and spasticity. These clinical findings align with our observations. However, despite the increasing utilization of acupuncture, its mechanisms in treating CP remain ambiguous, limiting broader clinical application. Manual acupuncture (MA) and electroacupuncture (EA) are the two primary methods of acupuncture, with EA favored for its standardized stimulation parameters, ensuring reproducibility in basic research. Our study employs EA to explore partial mechanisms in a rodent model of CP, using a 10 Hz continuous wave stimulation protocol consistent with previous literature.^21^ We have done preliminary trials to optimize EA parameters, confirming that 1mA stimulation effectively modulated responses without inducing discomfort. Unlike previous studies using anesthetized rodents,^21^ ours involved conscious animals to avoid anesthesia‐related cognitive impacts, thereby closely mirroring clinical scenarios where acupuncture is administered to awake patients.

Our study revealed that post‐EA treatment, there was a significant improvement in both motor and cognitive functions in juvenile rats with CP. Transcriptomic analysis indicated that these enhancements may be attributed to the activation of the PPAR pathway, particularly observed in the motor cortex epithelial cells of EA‐treated young rats with CP. This activation was further confirmed through qPCR analysis. Additionally, administering the PPAR agonist Rosiglitazone (Ros) exacerbated the CP phenotype, establishing a causal link between PPAR pathway activation and phenotypic improvement in CP. Our research represents the first transcriptomic comparison of gene expression post‐EA treatment in a CP rat model. Through this analysis, we discovered that EA alleviates the CP phenotype via the PPAR pathway, findings corroborated by qPCR and supported by the efficacy of Ros. Despite the absence of prior reports on Ros's application in CP, our findings uncover a novel molecular mechanism underlying EA's effectiveness in treating CP syndrome, potentially offering an alternative therapeutic approach for pediatric CP patients.^22^

Our research stems from clinical phenomena, innovatively exploring the mechanisms of EA treatment for CP from a novel perspective. While existing literature lacks elucidation on EA's mechanism in CP through the PPAR pathway, studies have reported PPAR, particularly PPAR‐γ, interventions in Alzheimer's disease,^23^ ischemia–reperfusion injury,^24^, ^25^ and other neurodegenerative disorders.^26^ These studies have played a theoretical role in supporting our discovery that EA can also improve CP symptoms by activating the PPAR pathway, especially *PPAR‐γ*, indicating its potential for anti‐inflammatory and neuroprotective effects akin to those observed in Alzheimer's disease and other neurological conditions.

Ros, a member of the thiazolidinedione class of antidiabetic medications, is a potent and highly selective agonist of the *PPAR‐γ*. Belonging to the nuclear hormone receptor superfamily, Ros activates the *PPAR‐γ* nuclear receptor, modulating the transcription and expression of various target genes. Its capabilities extend beyond glycemic control to include anti‐inflammatory effects,^27^ free radical scavenging, antioxidative stress responses, anti‐atherosclerosis, and anti‐tumor activities.^28^, ^29^, ^30^ Despite extensive researches into the therapeutic applications of Ros for neurodegenerative diseases,^26^, ^31^ its potential application in treating CP remains vacant. Ischemic–hypoxic brain injury, a primary pathological feature of CP, involves brain tissue changes due to partial or complete lack of oxygen, reduced or halted cerebral blood flow.^32^ Neuroinflammatory responses play a crucial role in this process, releasing numerous pro‐inflammatory factors and lipid peroxidation products, leading to neuronal death, delayed brain cell differentiation, and myelination, ultimately progressing to CP.^33^ Therefore, finding effective means to suppress post‐ischemic–hypoxic brain inflammation and promote neural functional recovery is key to treating CP. Current research indicates that Ros's primary mechanism in treating neurodegenerative diseases is through inflammation suppression for neuroprotection.^34^, ^35^ This study, for the first time, demonstrates that acupuncture can activate the PPAR pathway to improve CP symptoms, proposing the potential application of the PPAR agonist Ros in CP treatment.

In conclusion, this study provides molecular insights into EA treatment for CP, linking changes in the PPAR pathway within the motor cortex to improvements in CP symptoms. However, we have only demonstrated the mechanism of improving CP by EA from a single perspective. The mechanism of therapeutic effect of different acupuncture points and stimulation parameters is very complex, and PPAR may be only one of the pathways that play an important role. Further and more comprehensive studies are needed to clarify the molecular mechanism of improving CP symptoms by EA.

## 5 CONCLUSION

In summary, this study is the first to demonstrate that acupuncture can improve symptoms of CP through the activation of the PPAR pathway, encompassing enhancements in motor skills and cognitive functions. Furthermore, the use of the PPAR pathway agonist, Ros, in this research establishes a causal relationship between PPAR pathway activation and the amelioration of CP symptoms. This finding suggests the potential applicability of Ros in the treatment of CP, providing novel insights and research directions for therapeutic approaches to this condition.

### 5.1 Limitations

Our study demonstrates the potential of electroacupuncture to activate the peroxisome proliferators‐activated receptor pathway and improve the symptoms of cerebral palsy in a murine model. However, it is important to recognize that the CP condition induced in murine may not accurately reflect the full range of symptoms seen in pediatric patients with CP, including orthopedic limitations such as torsional deformities, muscle weakness, and hypertonia. Therefore, caution should be taken when extrapolating these findings to the clinical setting due to the complexity of the CP phenotype. Future investigations should encompass a more comprehensive evaluation of these clinical metrics to gain deeper insights into the effects of electroacupuncture on CP.

## AUTHOR CONTRIBUTIONS

Zhi‐Feng Wu and Yu‐Ping Zhang designed the study; Zhi‐Feng Wu, Hong‐hao Peng, Yun Shu, Li Zhang, Si Zhang, Jing‐yang Zhang, Si‐jie Li, Qiong‐li Fan, Yun Wei, Li Ming, and Jing‐jing Tong performed the experiments, analyzed the data, and wrote the manuscript.

## FUNDING INFORMATION

This article was supported by the National Natural Science Foundation of China (NO82104696) and the Natural Science Foundation of Chongqing (CSTB2023NSCQ‐MSX0266).

## CONFLICT OF INTEREST STATEMENT

The authors have no conflicts of interest to declare.

## PATIENT CONSENT STATEMENT

This research was conducted as an animal study. It did not involve human subjects; therefore, a patient consent statement is not applicable for this research. All procedures performed in studies involving animals were in accordance with the ethical standards of the institution or practice at which the studies were conducted.

## PERMISSION TO REPRODUCE MATERIAL FROM OTHER SOURCES

This study did not involve the reproduction of material from other sources. All content presented in this research is original and is created by the authors.

## CLINICAL TRIAL REGISTRATION

This research does not constitute a clinical trial. As such, it was not registered as a clinical study in any trial registry. This study was conducted as a non‐clinical, experimental research.

## Supporting information


Figure S1.

## Data Availability

The original datasets generated and analyzed during the current study are available from the corresponding author on reasonable request. Interested parties may contact the corresponding author via email to access the data.
