# Supplementary material for: Electroacupuncture activates the peroxisome proliferators‐activated receptor pathway to improve the phenotype of cerebral palsy
Source: CNS Neurosci Ther. 2024 Jul 25;30(7):e14876. doi: 10.1111/cns.14876 (PMC11269887; doi:10.1111/cns.14876)
Supplement: Supplementary file 1 — Figure S1. [file CNS-30-e14876-s001.doc]

Supplementary Figure


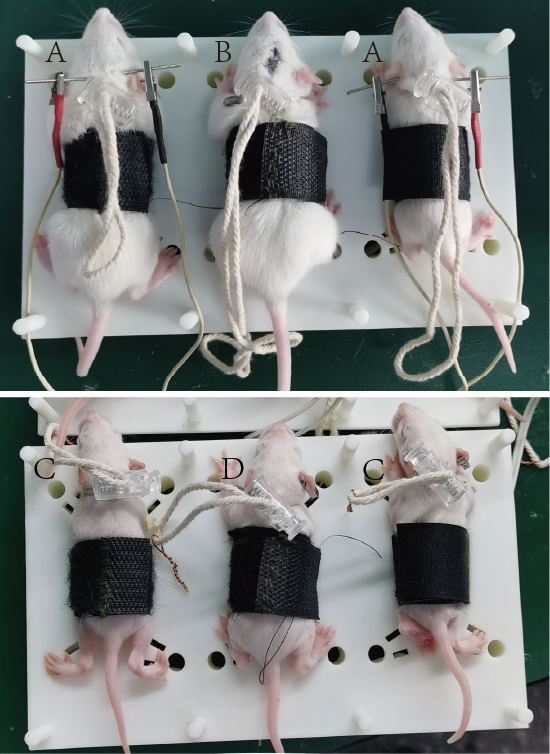


**Supplementary Figure**：To ensure consistent stress levels with EA, rats in all groups underwent the same immobilization procedure. Group A received EA treatment, Group B underwent sham operation, Group C served as the control group for CP, and Group D was the normal control group. (A-B represents postnatal day 21 in SD rats, while C-D represents postnatal day 14)
